# Bridging urology and palliative care: a narrative review of current practice and evolving priorities

**DOI:** 10.1097/MOU.0000000000001384

**Published:** 2026-03-18

**Authors:** Andreas Banner, Stephan Madersbacher, Lee A. Hugar, Eva K. Masel

**Affiliations:** a Department of Urology, Klinik Favoriten, Vienna; b Department of Social and Preventive Medicine, Center for Public Health, Medical University of Vienna; c Sigmund Freud Private University, Vienna, Austria; d Department of Genitourinary Oncology, H. Lee Moffitt Cancer Center, Tampa, USA; e Division of Palliative Medicine, Department of Medicine I, Medical University of Vienna, Vienna, Austria

**Keywords:** end of life, genitourinary cancer, narrative review, palliative care, urology

## Abstract

**Purpose of review:**

This narrative review summarizes recent evidence (2023–2025) on the role of palliative care in urology. It focuses on utilization trends, access disparities, symptom burden, procedural palliative needs, and end-of-life (EOL) care in advanced genitourinary malignancies.

**Recent findings:**

PC utilization in genitourinary cancers is increasing. Inpatient palliative care use rose from 4.9 to 31.5% in renal cell carcinoma and from 5.9 to 19.0% in testicular cancer. Yet, integration into routine care remains inconsistent. Disparities persist, including lower palliative care utilization among certain ethnic groups and uninsured patients, and higher odds of receiving high-intensity EOL care among socioeconomically vulnerable populations. Symptom burden remains substantial, with up to 82% of patients reporting multiple symptoms. This underscores the need for more timely palliative care involvement and more standardized reporting. Communication gaps, particularly regarding prognosis and EOL preferences, highlight opportunities for urologists to engage more actively in initiating palliative care discussions. Procedural palliative needs, including malignant ureteral obstruction management, illustrate the direct impact of urologists in providing symptom-directed palliative care.

**Summary:**

Despite growing recognition of palliative care gaps remain in access, referral timing, and systematic assessment. Addressing disparities, enhancing interdisciplinary collaboration, and prioritizing prospective research will be essential to improve patient and caregiver-centered outcomes.

## INTRODUCTION

Palliative care is now recognized as crucial for the management of urologic conditions - especially advanced genitourinary cancers given increasingly complex clinical, psychosocial and public health challenges that extend curative treatment [1,2]. Palliative care is specialized medical care for people living with chronic or serious illnesses, aimed at alleviating the symptoms and stress of the illness for both the patient and their caregivers [3]. Use of palliative care principles provides an extra layer of support, and is appropriate for patients of any age and at any stage of a serious illness. Hospice care represents a specific form of palliative care reserved for patients with limited life expectancy. Although no universal consensus definition of hospice exists, it generally differs from broader palliative care in that life-prolonging treatments are typically discontinued at the time of enrollment [2]. By contrast, palliative intent refers to the goal of a specific treatment or intervention aimed at prolonging survival while maintaining or improving quality of life, rather than achieving cure [4]. In this context, it is important to note that many palliative care studies rely on large administrative datasets, such as cancer registries or insurance claims data. These sources are susceptible to misclassification and may not reliably distinguish specialized palliative care from palliative-intent treatments, thereby limiting interpretability [4].

Recognition of palliative care in the urologic community is particularly timely given ongoing demographic shifts and a peak in incidence of genitourinary cancers after age 65. For example, the EU population aged 65 years and older is expected to increase significantly from 90.5 million in 2019 to 129.8 million in 2050 [5,6]. Additionally, three-quarters of adults in the United States live with at least one chronic condition, with more than half reporting 2 or more [7].

An aging population and an increase in patients with multiple complex comorbid conditions, creates a public health imperative to ensure equitable access to palliative care services. The public health paradigm is already shifting to emphasize population-level frameworks for treating serious illnesses, integration of advanced care planning, and alignment end-of-life (EOL) services with patient preferences and system sustainability [8,9]. Embedding palliative and EOL care into urology practice presents a promising but under-explored opportunity to advance patient-centered outcomes, reduce unnecessary health-care utilization, and address disparities in care delivery [2,10,11]. Unfortunately, access to and use of palliative care in the urologic community is lacking.

The aim of this narrative review is to explore the role of palliative care in urology by focusing on literature published within the past 2 years. We will examine how recent studies address the following: epidemiology and service use patterns in advanced urologic disease, symptom burden and quality of life, and care provided during the EOL period. Through this examination, we aim to elucidate key opportunities to increase the use of palliative care in urology, highlighting how cross-disciplinary collaboration can improve outcomes for patients and caregivers alike. 

**Box 1:**
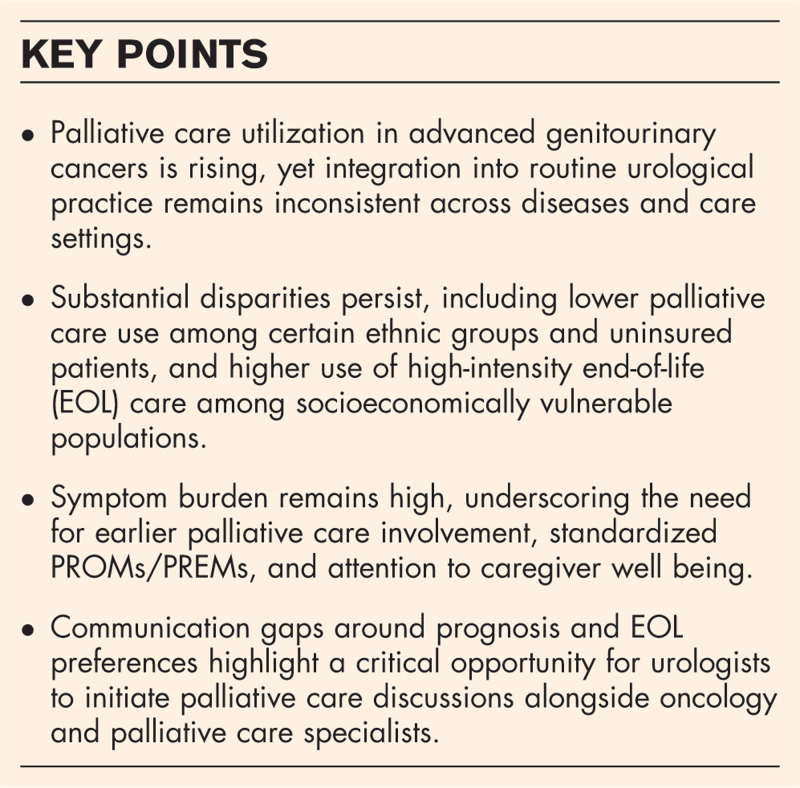
no caption available

## MATERIALS AND METHODS

**FIGURE 1:**
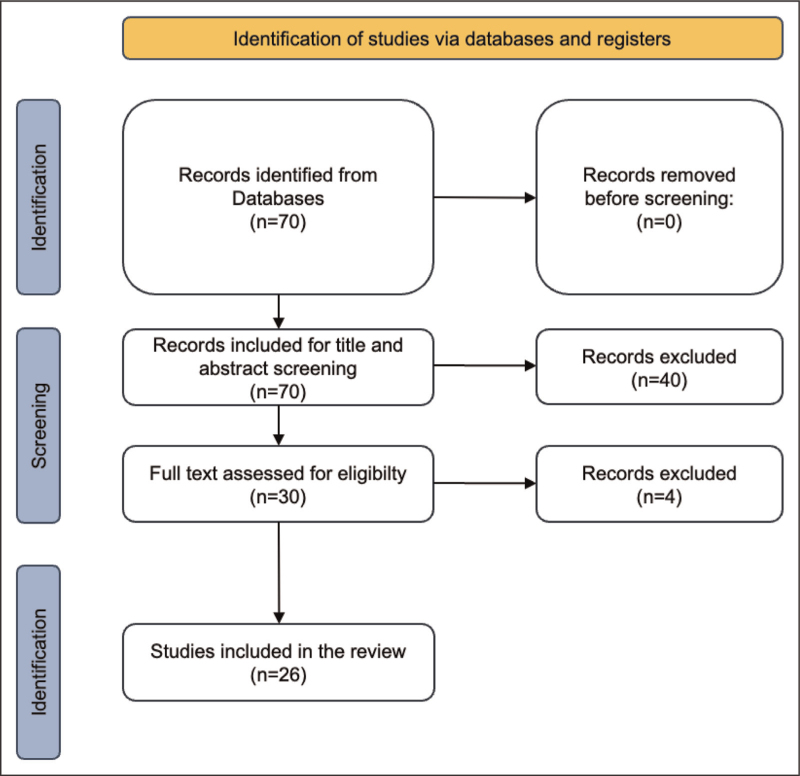
PRISMA Flow diagram for study selection.

One author (A.B.) performed a structured literature review using a predefined search strategy. The MeSH terms used are listed in the supplementary material. The review focused on publications from January 1, 2023, to October 1, 2025. A.B. screened titles and abstracts to identify suitable publications for inclusion. The co-authors suggested additional publications for inclusion. Figure 1 presents the PRISMA flow diagram for study selection. Graphical presentations of PC trends were generated using R (version 4.4.1). Data were aggregated from the included studies, and no original data collection or reanalysis was performed for this narrative review.

## RESULTS

### Trends and disparities

**FIGURE 2:**
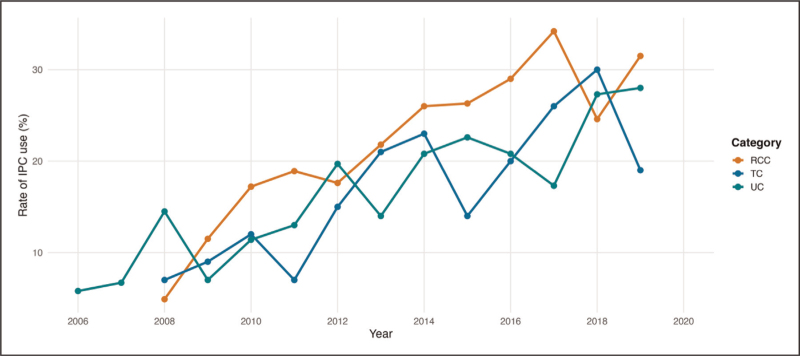
Temporal trends in inpatient palliative care use among patients with advanced renal cell carcinoma, testicular cancer, and urethral carcinoma based on National Inpatient Sample data [19,22,23].

Timely integration of palliative care into standard oncology care improves physical well being, patient satisfaction, and quality of care, and is recommended by international oncology guidelines [12–17]. Despite this, palliative care utilization among patients with advanced genitourinary cancers has historically been low (12.5–19.9%) [10]. Recent retrospective studies, however, suggest increasing palliative care uptake across multiple genitourinary malignancies [18,19,20^▪^,^21^–^23^]. A graphical summary for studies providing crude numbers is presented in Fig. 2.

Olafimihan *et al.* [21] reported a significant rise in palliative care consultations among hospitalized patients with metastatic prostate cancer, from 8490 to 15 231 per 100 000 admissions between 2010 and 2019. Similarly, relying on the National Inpatient Sample, Siech *et al.* [22] and Cano Garcia *et al.* [19] observed increasing inpatient palliative care (IPC) use through 2019 in patients with renal cell carcinoma (4.9–31.5%) and testicular cancer (5.9–19.0%). Additional studies showed rising palliative care utilization in metastatic upper tract urothelial cancer and urethral carcinoma [18,23]. Notably, studies in renal cell carcinoma and testicular cancer examined highly selected populations receiving critical care therapies (e.g., invasive ventilation, acute dialysis, or parenteral nutrition), and all identified analyses were based on U.S. data.

Beyond geographic variation in palliative care utilization [21–24], several studies have identified individual-level and system-level disparities. Eom *et al.* [24] found lower use of integrated care services within 12 months of advanced bladder cancer or renal cell carcinoma diagnosis among patients grouped as “other” ethnicities. In prostate cancer, Garg *et al.* [20^▪^] and Kohli *et al.* [25^▪^] reported lower palliative care use among specific ethnic subgroups, including Mexican Hispanics, Japanese, and Native Hawaiians. Conversely, Cano Garcia *et al.* [19] observed higher IPC rates among African–American testicular cancer patients in the U.S., likely reflecting more advanced disease and poor prognosis rather than improved access [26,27]. Studies that failed to detect disparities may have been limited by insufficient ethnic subgroup differentiation [18,22].

Broader equity considerations in urologic palliative care remain underexplored. Structural and social determinants of health – including socioeconomic status, rural residence, health literacy, mental illness, and social support – are likely to influence access to timely palliative care referral and service utilization. Barriers may include limited availability of specialized palliative care services in rural or resource-constrained settings, fragmented referral pathways, language and communication barriers, and differences in clinician referral practices. In the study by Olafimihan *et al.* [21], higher socioeconomic status, reflected by increased median household income, was associated with greater odds of receiving palliative care [quartile 1: reference; quartile 4: odds ratio (OR) 1.10, 95% confidence interval (95% CI) 1.03–1.18] [21]. Additionally, prostate cancer patients admitted to larger hospitals (OR 1.10, 95% CI 1.07–1.23), urban hospitals (OR 1.17, 95% CI 1.06–1.29) and teaching hospitals (OR 1.41, 95% CI 1.32–1.49) had higher odds of palliative care utilization. Similarly, Siech *et al.* [22] reported lower odds of IPC use among patients with renal cell carcinoma treated at smaller hospitals (OR 0.74, 95% CI 0.57–0.97), alongside increased odds in teaching hospitals (OR 1.41, 95% CI 1.17–1.71). In contrast, no association between IPC utilization and hospital size or teaching status was observed among patients with urethral carcinoma [23].

Additional disparities have been identified across care settings. Connors *et al.* [18] reported lower odds of palliative care utilization among patients with upper tract urothelial cancer treated at minority-serving hospitals (OR 0.70, 95% CI 0.51–0.96). A scoping review by Tamirat *et al.* [28^▪^] further highlighted care disparities among prostate cancer patients from culturally and linguistically diverse (CALD) backgrounds, citing a perceived lack of cultural sensitivity as contributing factor[29,30]. Finally, two studies identified disparities in palliative care utilization by insurance status, with uninsured patients being disadvantaged [24,31].

### Symptoms and quality of life

Patients with advanced, incurable cancer often experience worsening physical and psychological symptoms due to disease progression and treatment. Volberg *et al.* [32] reported a high symptom burden in patients with advanced genitourinary cancers, with 82% experiencing up to four symptoms and 18% reporting more than five. The most frequently reported symptoms were pain (61.4%), sleep disturbance (50.0%), diarrhea/constipation (42.9%) and sexual problems (31.4%). In the prospective PROCEED study, Rönningås *et al.* [33^▪▪^] observed 134 men with advanced prostate cancer receiving life-prolonging therapy and found a mean of 10 symptoms at baseline, increasing to 12 at last follow-up. Symptoms were most commonly related to androgen-deprivation therapy, including fatigue, sweating, and sexual problems.

Urinary obstruction is also common, occurring in approximately 13% of patients with advanced prostate cancer [34]. While androgen-deprivation seems to alleviate retention in most patients within 3 months (47.5–96.4%), upfront surgical deobstruction may be required in selected cases or with local disease progression [34–37]. A meta-analysis by Rosales *et al.* [38^▪▪^] demonstrated that palliative transurethral resection of the prostate (TUR-P) improves postvoid residual volume, symptom scores, and urinary retention but is associated with notable postoperative complications (repeat TUR-P in 23.4%, incontinence in 13.9%, and long-term catheterization in 10.8%). A small propensity-matched study identified both TUR-P and holmium laser enucleation (HoLEP) as feasible palliative treatment options [39]. In another small comparative study, Xu *et al.* [40] reported superior long-term symptom control and safety with palliative holmium laser enucleation compared with plasma kinetic prostate resection.

Palliative care may be delivered in inpatient or ambulatory settings, depending on symptom burden and care pathways. Degener *et al.* [41^▪▪^] conducted a near-population-based longitudinal study of genitourinary cancer patients (*n* = 5125) receiving specialized ambulatory palliative care (SAPC). At baseline, functional impairment was substantial (Karnofsky score ≤40% in 79.6%; ≤30% in 56%). Over time, symptoms such as appetite loss and vomiting remained stable, while dyspnea and pain improved significantly. Despite increased fatigue, overall functional status improved, as reflected by higher Karnofsky scores.

Inpatient palliative care has also demonstrated benefit in severe symptom settings. Dörr *et al.* [42] reported significant symptom relief and improved psychological well being among patients with renal cell carcinoma admitted for IPC, based on sequential Minimal Documentation System (MIDOS) and distress thermometer assessments. In a single-center interventional study, Xie *et al.* [43] found that patients with bladder cancer referred to early palliative care experienced greater improvements in quality of life (QoL), pain, fatigue, and anxiety compared with those receiving conventional care alone.

Psychological distress in advanced cancer patients may be exacerbated by limited disease understanding. In the study by Xie *et al.* [43], early palliative care explicitly included assessment of patients’ understanding of their illness. Supporting this, Heuser *et al.* [44] found that 29.5% of patients with advanced cancer were unaware they were receiving palliative treatment. Free-text responses in a survey by Johnson *et al.* [45] further highlighted deficiencies in communication, symptom management, and care coordination. Ensuring accurate, ongoing assessment of patients’ understanding of their disease and prognosis is essential for shared decision-making and high-quality palliative care [46].

### End-of-life care

End-of-life care aims to manage distressing symptoms comprehensively and to facilitate open communication about impending death from incurable disease [47]. While there is overlap between palliative care and hospice care, hospice typically emphasizes psychological support and administration of palliative care during the EOL period. In some countries, the terms palliative care and hospice are used interchangeably, whereas in other countries, hospice specifically refers to the cessation of curative treatments while continuing to support comfort and quality of life [48].

Because individual survival cannot be precisely predicted, the EOL period is variably defined, commonly ranging from 3 to 12 months. High-intensity EOL care – an established indicator for palliative care quality defined by Earle *et al.* [49] – is a composite measure that includes one or more of the following: ICU admission, multiple hospitalizations, or emergency department visits during the last month of life, or chemotherapy within the final 2 weeks of life or in-hospital death. Baird *et al.* [31] performed a retrospective SEER analysis to identify predictors of high-intensity EOL care across multiple cancers, including prostate cancer. High-intensity EOL care occurred in 38.6% of patients, with ICU admission in the final month being the most prevalent indicator. Regional variation existed, and higher comorbidity increased the likelihood of high-intensity care, whereas advanced age and poorer performance status were associated with lower risk.

The ability to express a preferred place of death represents an important aspect of patient autonomy and is shaped by social, spiritual, and medical factors [50,51]. A systematic review and meta-analysis found that 55% of patients preferred to die at home, 17% in hospital, and 10% hospice settings [52]. However, these estimates may vary by regional, cultural, and individual contexts [32,53]. A near-population-based study by Banner *et al*. showed higher in-hospital mortality among prostate cancer patients younger than 70 years (75.0%) compared with those aged at least 70 years (61.7–66.6%). Mellgård *et al.* similarly reported higher in-hospital mortality in patients with better performance status (ECOG < 2: 56.1%) versus those with ECOG ≥2 (35.7%). Notably Degener *et al.* observed an in-hospital mortality of only 8.6% among patients receiving SAPC, underscoring its potential to support EOL care aligned with patient preferences.

## DISCUSSION

In line with recent frameworks that advocate for the earlier integration of palliative care in urology, we observed an increasing trend in palliative care utilization among patients with genitourinary cancers. This reflects a shift away from using palliative care as a “last resort” [1,2,17]. Unfortunately, the rising incidence of genitourinary malignancies poses a growing challenge to ensuring equitable access to palliative care services [6,54]. Substantial gaps in palliative care uptake and timely integration persist, highlighting opportunities for public health-oriented strategies to identify, monitor, and address system-level barriers [10,11]. Clinical triggers that may guide timely palliative care referral are presented in Table 1[2].

**Table 1:** Clinical triggers for timely referral to specialized palliative care

1. High healthcare resource utilization
a. Frequent emergency department visits (≥2 in a month)
b. Any ICU-level care due to multiorgan system failure
2. Persistent pain or high risk of poor pain management
a. Neuropathic pain
b. Incident or breakthrough pain
c. Pain with severe associated psychosocial or family distress
d. Rapid escalation of opioid dose
e. Multiple allergies or adverse reactions to pain medications
f. Concerns regarding substance abuse disorder
3. High nonpain symptom burden or symptoms refractory to initial management
a. Anorexia and/or cachexia, nausea and vomiting, constipation, and diarrhea
b. Fatigue, weakness or asthenia, insomnia or sedation, delirium
c. Dyspnea
d. High distress
e. Lymphoedema
f. Hormone-related
4. Limited anticancer treatment options
a. Limited access to healthcare resources
b. Advanced-stage disease
c. Severe or multiple comorbidities
d. Rapidly declining or poor functional status
5. Need for advanced communication skills
a. Resistance to engage in advanced care planning
b. Need for clarification of goals of care
c. Assessment of decision-making capacity
d. Communication barriers (language, literacy, cognitive impairment)
e. Patient request for hastened death
6. Complex patient and/or caregiver circumstances
a. High risk, or presence, of complex bereavement disorder
b. Inadequate social support
c. Substance use
d. Financial limitations or financial toxicity
e. Discordant expectations or goals of care
7. Oncology care challenges
a. Complex care coordination issues or involvement of multiple care teams
b. Intra-team conflict
c. Burnout and/or compassion fatigue
d. Moral distress or ethical concerns

Reprinted and adapted from the study by Hugar *et al.* with permission from Springer Nature [2].

Palliative care and EOL care are recognized as human rights in international guidelines, yet access remains influenced by healthcare availability and insurance status [1]. Although existing studies suggest potential racial disparities in the receipt of palliative care services, these findings may be confounded by unmeasured differences in tumor aggressiveness and disease stage. Two studies detected differences in palliative care service utilization among patients with different insurance status, with the lowest among those uninsured [24,31]. Escalating healthcare costs – particularly in the final months of life – contribute to increasing financial toxicity, which may further restrict access to palliative care services [55,56]. Conversely, Baird *et al.* [31] reported higher odds of receiving high-intensity EOL care among prostate cancer patients whose healthcare costs were fully covered by the state. Collectively, these findings highlight that socioeconomic vulnerability, whether through lack of insurance or complete reliance on state-funded coverage, may shape markedly different and often suboptimal trajectories of EOL care.

Symptom burdens have been evaluated in only a subset of included studies and may not fully reflect real-world clinical complexity. Comparability across studies is further limited by heterogenous assessment tools. Future research should prioritize validated patient-reported outcome and experience measures (PROMs/PREMs) to standardize symptom assessments and longitudinal follow-up. The integration of electronic PROMs/PREMs represents a promising opportunity to facilitate ongoing monitoring. Importantly, caregiver wellbeing should also be addressed, as Johnson *et al.* [46] reported that 86% of caregivers had unmet needs related to their caregiving capacity or personal wellbeing.

Communication of palliative and EOL treatment goals should not be limited to palliative care physicians or medical oncologists; urologists should be encouraged to actively engage in the early palliative care process. A recent qualitative study involving urologists, palliative care physicians, and multidisciplinary team members identified several opportunities to facilitate this integration [57]. Early patient education regarding prognosis and treatment goals supports timely incorporation of palliative measures, including discussions of individual preferences and EOL wishes. Notably, Volberg *et al.* [32] reported that 71% of patients had never been asked about their EOL preferences, and only 25% had been approached by their urological oncologist about treatment and care options, indicating a vital opportunity to improve patterns of care.

Urology's role in initiating palliative care discussions is not limited to patients with genitourinary malignancies, as other cancers may cause urological complications such as malignant ureteral obstruction (MUO). Although advances in endoscopic ureteral stent placement have reduced the need for percutaneous nephrostomy (PCN), stenting remains prone to failure due to anatomical and technical limitations in this population [58]. Moreover, PCN is frequently associated with complications, including infections and dislodgement, which compromises comfort at the EOL. In a study by Felice *et al.* [59^▪▪^], only 39.1% of patients were referred to PC before or upon diagnosis of MUO, whereas 80% were referred to urology consultation, indicating a potential role of urology for PC initiation upon discussion of invasive treatment options for MUO.

Although most included studies used reproducible and well defined methodologies, all were subject to limitations. Several analyses were retrospective and focused on highly selected populations, limiting generalizability of their findings. In addition, many study periods predate recent advances in genitourinary oncology, further constraining applicability to contemporary patient populations. Furthermore, most studies relied on US-based data, which may reduce international relevance. A key limitation is the reliance on retrospective registry data, in which palliative care utilization was often identified using nonspecific coding (e.g., ICD-10-PCS) or vague definitions. This approach is prone to misclassification, as codes may capture other palliative-intent interventions – such as first-line palliative systemic therapy or palliative surgery – rather than specialized palliative care services provided by a multidisciplinary palliative care team. Patient-centered and qualitative studies were scarce, limiting insights into patient experiences, preferences, symptom burden, and perceived care needs. Finally, nononcologic urologic palliative care needs remain underrepresented in contemporary research and warrant further investigation.

## CONCLUSION

Palliative care is increasingly recognized as a core component of urologic oncology, yet its integration into routine practice remains inconsistent. Although utilization has risen in recent years, substantial disparities persist by race, insurance status, and structural access barriers. The high symptom burden experienced by patients underscores the need for more timely palliative care involvement, routine use of standardized PROMs and PREMs, and greater support for caregivers. Persistent communication gaps, particularly around prognosis and EOL preferences, highlight missed opportunities for urologists to engage more proactively in palliative care discussions. Future research should refine palliative care definitions, address inequities in access, and evaluate care models that mitigate financial toxicity. Meaningful integration of palliative care into urologic practice has the potential to improve patient-centered outcomes and better align EOL care with individual values.

## Acknowledgements

*Manuscript writing and editing: A. Banner*

*Supervision and oversight: E. Masel, S. Madersbacher, L. Hugar*

*The manuscript has been seen, reviewed, and approved by all contributing authors.*

*During the preparation of this work, the first author used Rubriq for copyediting. All outputs were reviewed and verified by the first author, who takes full responsibility for the final content of the publication.*

### Financial support and sponsorship

*No funding was used for the creation of this review.*

### Conflicts of interest

*The authors declare that they have no competing interests.*

## Supplementary Material

Supplemental Digital Content

## REFERENCES AND RECOMMENDED READING

Papers of particular interest, published within the annual period of review, have been highlighted as:

▪ of special interest
▪▪ of outstanding interest
