# Supplementary material for: Bridging urology and palliative care: a narrative review of current practice and evolving priorities
Source: Curr Opin Urol. 2026 Mar 18;36(4):423–30. doi: 10.1097/MOU.0000000000001384 (PMC13258080; doi:10.1097/MOU.0000000000001384)
Supplement: Supplemental Digital Content [file couro-36-423-s001.docx]

**Bridging Urology and Palliative Care: A Narrative Review of Current Practice and Evolving Priorities**

Andreas Banner ^1,2^, Stephan Madersbacher^1,3^, Lee A. Hugar^4^, Eva K.Masel^5^

^1^ Department of Urology, Klinik Favoriten, Vienna, Austria ^2^ Department of Social and Preventive Medicine, Center for Public Health, Medical University of Vienna, Austria ^3^ Sigmund Freud Private University, Vienna, Austria ^4^ Lexington Urology, Lexington Medical Center, West Columbia, SC, USA ^5^ Division of Palliative Medicine, Department of Medicine I, Medical University of Vienna, Vienna, Austria

**Search strategy:**

cancer, urological; cancer, urologic; cancer, genito urinary; cancer, genitourinary; cancer, prostate; cancer of the prostate; cancer of prostate; cancer, urinary bladder; cancer, urinary tract; cancer, renal; cancer, renal cell; cancer, testicular; cancer, testis; cancer, penile; cancer, penis; cancer, ureteral; cancer, ureter; cancer, urethral; cancer, urethra **(OR)**

**(AND)**

Care, palliative; palliative care; hospice and palliative care nursing; medicine, palliative; medicine, palliative care; supportive care, palliative; care, hospice; hospice; hospice care; hospice nursing; care, end of life; end of life; end of life care **(OR)**

Search period: 2023-2025

Search platform: Pubmed (case-reports and non-peer-reviewed articles were excluded)

Initially identified: Pubmed (n=70); After title and abstract screening: Pubmed (n=30); After full text screening: Pubmed (n=26)
